# HCV Causes Chronic Endoplasmic Reticulum Stress Leading to Adaptation and Interference with the Unfolded Protein Response

**DOI:** 10.1371/journal.pone.0024660

**Published:** 2011-09-19

**Authors:** Emmanuelle Merquiol, Dotan Uzi, Tobias Mueller, Daniel Goldenberg, Yaakov Nahmias, Ramnik J. Xavier, Boaz Tirosh, Oren Shibolet

**Affiliations:** 1 Liver Unit, Division of Medicine, Hadassah-Hebrew University Medical Center, Jerusalem, Israel; 2 Institute for Drug Research, School of Pharmacology, Faculty of Medicine, Jerusalem, Israel; 3 Medizinische Klinik M. S. Hepatologie und Gastroenterologie, Charité -Universitätsmedizin Berlin, CVK, Berlin, Germany; 4 Goldyne Savad Institute of Gene Therapy, Hadassah-Hebrew University Medical Center, Jerusalem, Israel; 5 Center for Bioengineering, Rachel and Selim Benin School of Computer Science and Engineering, Silberman Institute of Life Sciences, The Hebrew University of Jerusalem, Jerusalem, Israel; 6 Gastroenterology Unit, Center for the Study of Inflammatory Bowel Disease, and Center for Computational and Integrative Biology, Massachusetts General Hospital and Harvard Medical School, Boston, Massachusetts, United States of America; 7 Liver Unit, Department of Gastroenterology and Liver Disease, Tel-Aviv Sourasky Medical-Center, Tel-Aviv, Israel; Ulm University, Germany

## Abstract

**Background:**

The endoplasmic reticulum (ER) is the cellular site for protein folding. ER stress occurs when protein folding capacity is exceeded. This stress induces a cyto-protective signaling cascades termed the unfolded protein response (UPR) aimed at restoring homeostasis. While acute ER stress is lethal, chronic sub-lethal ER stress causes cells to adapt by attenuation of UPR activation. Hepatitis C virus (HCV), a major human pathogen, was shown to cause ER stress, however it is unclear whether HCV induces chronic ER stress, and if so whether adaptation mechanisms are initiated. We wanted to characterize the kinetics of HCV-induced ER stress during infection and assess adaptation mechanisms and their significance.

**Methods and Findings:**

The HuH7.5.1 cellular system and HCV-transgenic (HCV-Tg) mice were used to characterize HCV-induced ER stress/UPR pathway activation and adaptation. HCV induced a wave of acute ER stress peaking 2–5 days post-infection, which rapidly subsided thereafter. UPR pathways were activated including IRE1 and EIF2α phosphorylation, ATF6 cleavage and XBP-1 splicing. Downstream target genes including GADD34, ERdj4, p58ipk, ATF3 and ATF4 were upregulated. CHOP, a UPR regulated protein was activated and translocated to the nucleus. Remarkably, UPR activity did not return to baseline but remained elevated for up to 14 days post infection suggesting that chronic ER stress is induced. At this time, cells adapted to ER stress and were less responsive to further drug-induced ER stress. Similar results were obtained in HCV-Tg mice. Suppression of HCV by Interferon-α 2a treatment, restored UPR responsiveness to ER stress tolerant cells.

**Conclusions:**

Our study shows, for the first time, that HCV induces adaptation to chronic ER stress which was reversed upon viral suppression. These finding represent a novel viral mechanism to manipulate cellular response pathways.

## Introduction

Hepatitis C virus (HCV) is a hepatotropic, positive-strand RNA member of the Flaviviridae family [1]. Approximately 170 million people worldwide are infected with HCV. A notable feature of the virus is its tendency to cause chronic infection. Only a minority of infected patients is able to eradicate the virus, and up to 80% of the patients become chronic carriers. The molecular mechanisms leading to viral persistency and its ability to evade intracellular host defenses are multi-fold and only partly understood [2]. Currently, there is no effective vaccine against HCV and anti-viral therapy has limited efficacy [3].

Similar to other positive-strand RNA viruses, HCV replication involves the formation of a membrane associated replication complex [4]. The origin of these membranes can be the mitochondria, intracellular organelles or the endoplasmic reticulum (ER). Various structural and non-structural HCV proteins were shown to localize inside the ER lumen or integrated to ER membrane and to undergo ER-specific modifications [5], [6].

The ER is the cellular site for folding and modification of membrane-bound and secreted proteins. The flux of newly synthesized proteins into the ER is dynamic. Conditions of ER stress occur when the amount of proteins entering the ER exceeds its folding capacity. This imbalance induces a cyto-protective signaling cascade collectively termed the unfolded protein response (UPR) [7], [8]. The mammalian UPR operates in several parallel pathways, whose sensors are IRE1, PERK and ATF6. When misfolded proteins accumulate in the ER, IRE1 is activated by trans-autophosphorylation and splices the mRNA of XBP-1, which converts the unspliced XBP-1 (XBP-1u), a highly unstable protein into its spliced form (XBP-1s). XBP-1s is a potent transcription factor responsible for the transcriptional activation of the majority of UPR target genes [9]. PERK, a second ER stress sensor, is activated in a similar fashion to IRE1 and is responsible for the modulation of protein synthesis in response to ER stress. Activated PERK phosphorylates eIF2α and leads to general suppression of protein synthesis and activation of the integrated stress response [10], [11]. ATF6, the third sensor, operates both as a stress sensor and as a transcription factor. In response to ER stress, ATF6 translocates from the ER to the Golgi apparatus, where its N-terminus is released from the membrane by regulated intramembrane proteolysis [12]. Activation of the UPR leads to a coordinated and highly regulated response which improves the efficiency of protein folding, processing and export. In order to reduce protein load in the ER, the UPR attenuates the flow of proteins into the ER and decreases protein synthesis, while up-regulating the expression of chaperones. Finally, it removes misfolded proteins via activation of ER associated degradation (ERAD).

The UPR is intimately linked to the apoptotic machinery. Both IRE1 and PERK, when strongly activated, lead to apoptosis [13], [14]. While acute ER stress conditions signal for cell death, chronic sub-lethal ER stress causes cellular adaptation and eventually resistance to apoptosis in a mechanism, which was suggested to involve modulation of mRNA localization and stability of UPR components [15].

It was previously shown that in cells stably expressing the HCV replicon, XBP-1 mRNA undergoes splicing, indicative of ER stress. However the expression of specific target genes downstream to XBP-1s responsible for enhanced degradation of misfolded ER proteins was not elevated. These results suggested that HCV manipulates the IRE1-XBP-1 pathway of the UPR. It was further shown that ER stress develops in response to expression of specific HCV proteins such as the core protein E1, E2, NS2 and NS4B [16]–[19]. Whether this response triggers apoptosis or pro-survival signals is unclear.

It is noteworthy that most of these studies were performed not in the context of viral infection, but rather by using transfections of sub-genomic or full-length replicons, as well as cell lines (hepatocytes and non-hepatocyes) over-expressing specific viral proteins. As readouts, these studies used mostly luciferase reporters and were limited to single arms of the UPR. Furthermore, most did not explore downstream targets of UPR activation or stated that disrupted activation of UPR exists [16]–[18], [20]–[22]. More recently, induction of ER stress was also demonstrated in the context of the HuH7.5.1 model, a cell culture system which supports a full replication cycle of HCV [23], [24]. All three pathways of the UPR were activated: PERK underwent phosphorylation, ATF6 was cleaved and XBP-1 mRNA was spliced. However, it was not conclusively shown whether the target genes of the various pathways were correspondingly activated. In contrast, Lerat et al, showed in a transgenic mouse model expressing the full replicon of HCV that ER stress genes are not activated [25]. In addition, Joyce et al, showed that infection of human hepatocytes by HCV in-vivo in the chimeric *SCID/Alb-uPA* mice resulted in enhanced Bip/Grp78 expression, but no significant activation of UPR target genes [26]. These data may suggest that the UPR was either partially activated or that its downstream signaling was suppressed or kept at a minimal level probably to avoid ER stress-mediated apoptosis. All of the studies were limited to the acute infection soon after virus introduction. However, chronic infection by HCV, rather than the acute phase is the major component of HCV pathology in humans.

In this study we explored whether HCV infection causes chronic long lasting ER stress conditions, and if so, is adaptation to continuous ER stress, which was demonstrated to occur in response to pharmacological ER agents develops. Using the HuH7.5.1 system we demonstrate that HCV induces the UPR in a wave-like fashion, which peaks on days 3–5 after infection. The UPR then subsides but remains active at a low level. This chronic ER stress conferred adaptation and resistance to further drug-induced ER stress. Suppression of viral replication using interferon-α 2a treatment restored UPR responsiveness, indicating that continues presence of the virus is required for adaptation. To address this phenomenon in vivo, we used mice expressing the HCV replicon in their liver (HCV-Tg). These mice displayed basal low level chronic ER stress and adaptation of UPR when administered tunicamycin, an ER stressor. These findings represent a novel mechanism by which chronic HCV infection may disrupt cellular homeostasis.

## Materials and Methods

### Cell Culture

Huh 7.5.1 cells, a kind gift from Dr. R Chung (Massachusetts General Hospital, Boston, MA, USA) [23], were grown in Dulbecco's modified Eagle's medium (DMEM) supplemented with 10% fetal calf serum, 100 units/ml penicillin, 100 µg/ml streptomycin and 2 mM-glutamine (Biological Industries, Israel). Cells were grown at 37°C in a 5% CO2 atmosphere within a humidified incubator.

### JFH1 system

Cell supernatant containing infectious JFH1 HCV 2a virus was harvested from day 7–20 and frozen at −80°C as previously described [27]. Culture media from Huh 7.5.1 (80% confluent) in 10 cm dish was removed and replaced by 3 ml of thawed JFH1 viral stock. After 3 h medium was replaced by DMEM. Titration of viral infection was done as previously described [28]. In brief, Huh7.5.1 cells were seeded in 96-well plates at 1×10^4^ cells/well. Supernatant was serially diluted 10-fold in complete growth medium and used to infect the seeded cells (6 to 8 wells per dilution). Following 2 or 3 days of incubation the cells were immunostained for HCV core protein. Wells that expressed at least one core expressing cell were counted as positive, The TCID_50_ was calculated using the TCID50 calculator http://www.med.yale.edu/micropath/pdf/infectivity%20calculator.xls as previously described [29]. Multiplicity of infection (MIO) was calculated using a conversion formula: http://www.bioon.com./book/biology/e-protocol/cell/miopfu.htm.

Quantification of HCV RNA by RT-PCR was done as previously described [27]. In brief: Total RNA was used to quantify HCV. JFH copy number was calculated according to a standard curve run in each RT-PCR experiment. The standard curve was calculated using a series of 10-fold dilutions of previously titrated JFH-1 plasmid. Titration of JFH-1 was done with Abbott RealTime HCV assay (Abbott, Illinois, USA), with previously published primers (Table 1). Viral quantization was done repeatedly throughout the experiments to ensure equal MIO. In order to avoid nutrient stress, cells were split when reaching approximately 80% confluence and growth media was regularly exchanged. Equal numbers of cells were used for each experiment.

**Table 1:** Primers used for real-time PCR.

Human:	
Actin-F	GCG GGA AATCGT GCG TGA CAT
Actin-R	GAT GGA GTT GAA GGT AGT TTC
ATF3-F	TTG CAG AGC TAA GCA GTC GTG
ATF3-R	ATG GTT CTC TGC TGC TGG GAT TCT
XBP1-spliced-F	CTG AGT CCG CAG CAG GTG
XBP1-spliced-R	TGC CCA ACA GGA TAT CAG ACT
XBP1-total-F	AGT AGC AGC TCA GAC TGC CA
XBP1-total-R	CCT GGT TCT CAA CTA CAA GGC
p58IPK-F	TTT GCG TTC ACA AGC ACT TAA C
p58IPK-R	GTT CTG CAT CCC AAA CAC AAA C
ERdj4-F	GGT GTG CCA AAA TCG GCA TC
ERdj4-R	GCA CTG TGT CCA AGT GTA TCA TA
GADD34-F	ATG ATG GCA TGT ATG GTG AGC
GADD34-R	AAC CTT GCA GTG TCC TTA TCA G

Mouse:	
p58IPK-F	TTTACTGCCGCAAGACTACAG
p58IPK-R	CTGGGGTTAGATTTGAGCACTT
ATF3-F	CAGACCCCTGGAGATGTCAGT
ATF3-R	TTCTTGTTTCGACACTTGGCA
ERdj4-F	ATAAAAGCCCTGATGCTGAAGC
ERdj4-R	GCCATTGGTAAAAGCACTGTGT
AFT4-F	CCTTCGACCAGTCGGGTTTG
AFT4-R	CTGTCCCGGAAAAGGCAT
UBC-F	CAGCCGTATATCTTCCCAGACT
UBC-R	CTCAGAGGGATGCCAGTAATCTA
XBP1 (Total)-F	AAGAACACGCTTGGGAATGG
XBP1 (Total)-R	ACTCCCCTTGGCCTCCAC
XBP1 (Spliced)-F	GAGTCCGCAGCAGGTG
XBP1 (Spliced)-R	GTGTCAGAGTCCATGGGA
CHOP-F	CTGGAAGCCTGGTATGAGGAT
CHOP-R	CAGGGTCAAGAGTAGTGAAGGT

XBP splicing	
XBP1-F	CCA TGG GGA GAT GTT CTG
XBP1-R	CCT GGT TGC TGA AGA GGA

JFH1	
F-JFH1: (58–77)	CTGTCTTCACGCAGAAAGCG
R-JFH1: (273-254)	TCGCAACCCAACGCTACTCG

### Mice

C57BL/6J HCV-Tg mice expressing the entire HCV 1b ORF under alpha-1 antitrypsin promoter were kindly provided by Prof. N. La Monica (IRBM, “P. Angeletti”, Pomezia, Italy) [30]. C57BL/6J wt mice (Harlan, Jerusalem, Israel) were used as controls. Animal care and experiments were approved and conducted in accordance with the Hebrew University-Hadassah Animal Authority guidelines (Ethical approval number: MD-1012389-5). Male mice, 10–12 weeks old, 24–26 gr were kept in the SPF unit in the animal facility at the Hebrew University Medical School. Mice were treated i.p with tunicamycin (Fermentek, Jerusalem, Israel) injection of 1 mg/kg (volume of 20 µl/1gr mouse) every other day (total 2–3), and were sacrificed 3 days following the last injection. Control mice received 5% dextrose injection in the same volume. Mice were anesthetized with Ketamine 5 mg/kg (Kepro, Holland) + Xylazine 5%, (Kepro), terminally bled from the heart and sacrificed by cervical dislocation. Liver samples, were immediately harvested into liquid nitrogen (and were kept in −80°C). A portion was fixed in 4% formaldehyde (Gadot, Israel) for 24 hrs, and then kept in 70% ethanol or in 30% sucrose.

### Reagents and antibodies

The following antibodies were used: NS5a (Virogen, MA, USA #276-A), XBP-1 (Santa Cruz CA, #7160), total-IRE1 α (Cell Signaling #3294) phospho-IRE1α (Abcam, Cambridge, UK, #48187), phospho/total- eiF2α (Cell Signaling #9721, #9722), BiP (Abcam #21685), ATF6 (Abcam #11909), CHOP (Santa-Cruz, #sc-575). Monoclonal mouse antibody against HCV core protein (Affinity BioReagents, CO, USA, #MA1-080), actin (Abcam #8227). Thapsigargin (Sigma, MO, USA), 1,000x stocks in DMSO.

### Western Blot Analysis

Cells were washed with PBS and lysed for 10 min on ice in Nonidet P-40 lysis buffer (50 mm Tris, 150 mm NaCl, 2 mm EDTA, 1% Nonidet P-40, 50 mm NaF, 1 mm Na3VO4, 10 mm Na2P2O4, protease inhibitor cocktail (Roche, IN, USA)). Lysates were cleared by centrifugation (14,000 rpm for 15 min at 4°C), protein concentration was determined and samples were boiled in reduced Laemmli sample buffer. For tissue samples, liver tissue was homogenized for 5 minutes with fresh homogenizing buffer (NaHCO_3_ 1 mM, CaCl_2_ 0.5 mM, 1% Protease Inhibitor and 1% Phosphatase inhibitor). Samples were centrifuged (2000 rpm for 5 min), and the pellet was treated with RIPA lysis buffer (NaCl 150 mM, NP-40 1%, DOC 0.5%, SDS 0.1% and TRIS 50 mM). Extracts were centrifuged (14,000 rpm for 10 min) protein concentration was determined and samples were boiled in reduced Laemmli sample buffer. Following SDS-PAGE of equal protein content, gels were transferred to a polyvinylidene difluoride (PVDF) membrane. Detection was performed using horseradish peroxidase-linked antibody and chemiluminescence (Santa Cruz). Membranes were probed with anti-actin to confirm equal loading.

### RT-PCR and quantitative RT-PCR

Total cellular RNA was isolated by Trizol reagent (Prio-Lab, Jerusalem, Israel) according to the manufacturer instructions. For reverse transcription, 0.5 µg of total RNA were transcribed using cDNA synthesis kit (Applied Biosystem, CA, USA). Aliquots of 1 µl cDNA were subjected to 35 cycles of PCR amplification. Actin or Ubc were used as internal control. The primer sequences are shown in table 1 (Each primer pair was designed to span an intron). Negative controls included the amplification of samples without prior RT reaction.

Quantitative RT-PCR was performed in the PCR 7900HT Real-Time PCR System (Applied Biosystems) using SYBR Green mix (Applied Biosystems). Briefly, 100 ng of reversed transcribed cDNA were used for each PCR reaction with 250 nM of forward and reverse primers. The thermal cycling conditions comprised 4 minutes at 95°C, followed by 35 cycles at 94°C for 30 seconds, 58°C for 5 seconds, and 72°C for 45 seconds. The mRNA level in untreated cells was defined as 1 arbitrary unit.

### Immunofluorescence

Cells were seeded in 24 well plates on cover glass. At specific time points the cells were washed three times with ice-cold PBS and fixed with 4% formaldehyde 0.2% Triton-X-100 at room temperature for 20 min. Cells were washed and incubated with PBS containing 5% donkey serum, 0.3% Triton X-100, and 10% FCS for 30 min. Cells were then incubated overnight with antibody to HCV core protein or CHOP, washed three times with PBS, and incubated with an anti-mouse antibody conjugated to Cy5 at a or anti-rabbit Alexa Fluor 488 conjugated (invitrogen, OR, USA).

### Interferon treatment and HCV quantification

Interferon α 2a (IFN-α 2a) (Roferon A, Roche, Basel, Switzerland) was used to suppress viral replication as previously described [31]. In brief: cells were infected with JFH-1 HCV 2a virus as described above. Following infection, 1800IU/ml of IFN-α 2a were added to cell growth medium and changed daily for the duration of the experiment. Quantification of HCV RNA was done by RT-PCR. Total RNA was used to quantify HCV. JFH1 copy number was calculated according to a standard curve that was done for each experiment. The standard curve was calculated using a series of 10-fold dilutions of previously titrated plasmid JFH1. Titration of plasmid JFH1 was performed with the Abbott RealTime HCV assay (Abbott, IL, USA).

### Immunohistochemical staining

Liver samples were fixed in 4% neutral-buffered formalin and embedded in paraffin. 5 µM sections were dewaxed and hydrated through graded ethanol dilutions, then cooked in citrate buffer (pH 6, 10 mM) in a pressure cooker at 115°C for 3 minutes. Endogenous peroxidase activity was blocked with 3% hydrogen peroxide followed by washing. The sections were then incubated with the indicated antibodies: CHOP (1∶300, Santa Cruz, SC-575). Secondary Ab exposure was performed using the Mach 3 Rabbit tool kit (Biocare Medical, CA, USA) following manufacturers' instructions. All sections were counterstained with hematoxylin, and were visualized using an Olympus light microscope. Quantification was performed by visualizing the slides on an Olympus BX61 microscope, photography was performed on Photometric CoolSnap ES and analysis was done using Ariol SL-50 Applied Imaging 3.1.270 software.

### Statistical analysis

Statistical significance between groups was assessed by one way ANOVA adjusted for multiple comparisons and tested for normal distribution. Data are expressed as means ± standard deviations (S.D.). Triplicate determinations were performed in all the real-time experiments, and all experiments were repeated at least three times. A p-value equal or less than 0.05 was considered to be statistically significant.

## Results

### HCV infection induces a wave like activation of the 3 arms of the UPR

To monitor the induction of UPR following infection by HCV we used the HuH7.5.1 cell system. Cells were infected with JFH1 2a strain and viral replication was assessed by RT-PCR for viral RNA and immunoblotting for the viral proteins NS5A and core. We observed high infectious titers and viral persistence up to 25 days post-infection. Following day 9 post-infection, we observed a steady-state level without further increases in viral replication. To ensure equal infection conditions in each experiment TCID50, MIO and HCV copy number were measured repeatedly as described in the methods (Figure S1a–b and data not shown). During culture, cells may display different levels of ER stress/UPR activation. In order to assess base line levels of ER stress we cultured non-infected cells in parallel to infected cells. We did not observe significant activation of ER stress in non-infected cells during days 1–5 of the experiment (Figure S3a).

**Figure 1:**
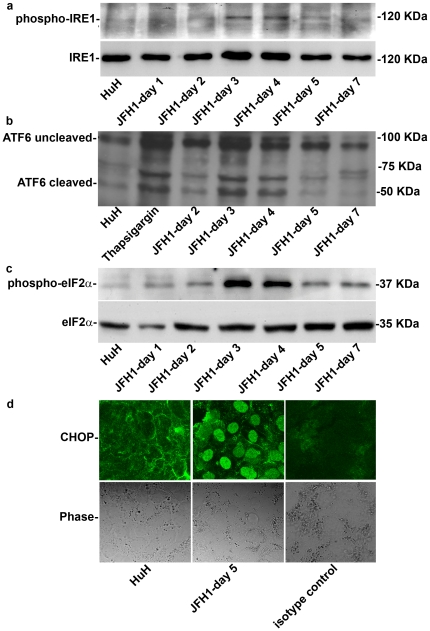
HCV infection induces a wave like activation of the 3 arms of the UPR. HuH7.5.1 hepatoma cells were infected with HCV-JFH1. Following infection, protein was extracted at the indicated time points. The expression of (a) phospho-IRE1, and (b) cleaved and non-cleavedATF6 (c) phospho-eIF2α were analyzed by western blotting. (d) CHOP translocation to the nucleus was assessed by immunofluorecence in control HuH7.5.1 cells or JFH1 infected cells on day 5, isotype anti-rabbit IgG was used as control. Results are means±S.D. of 3 experiments. Blots and picture are from a representative experiment (see also Figure S3a).

**Figure 2:**
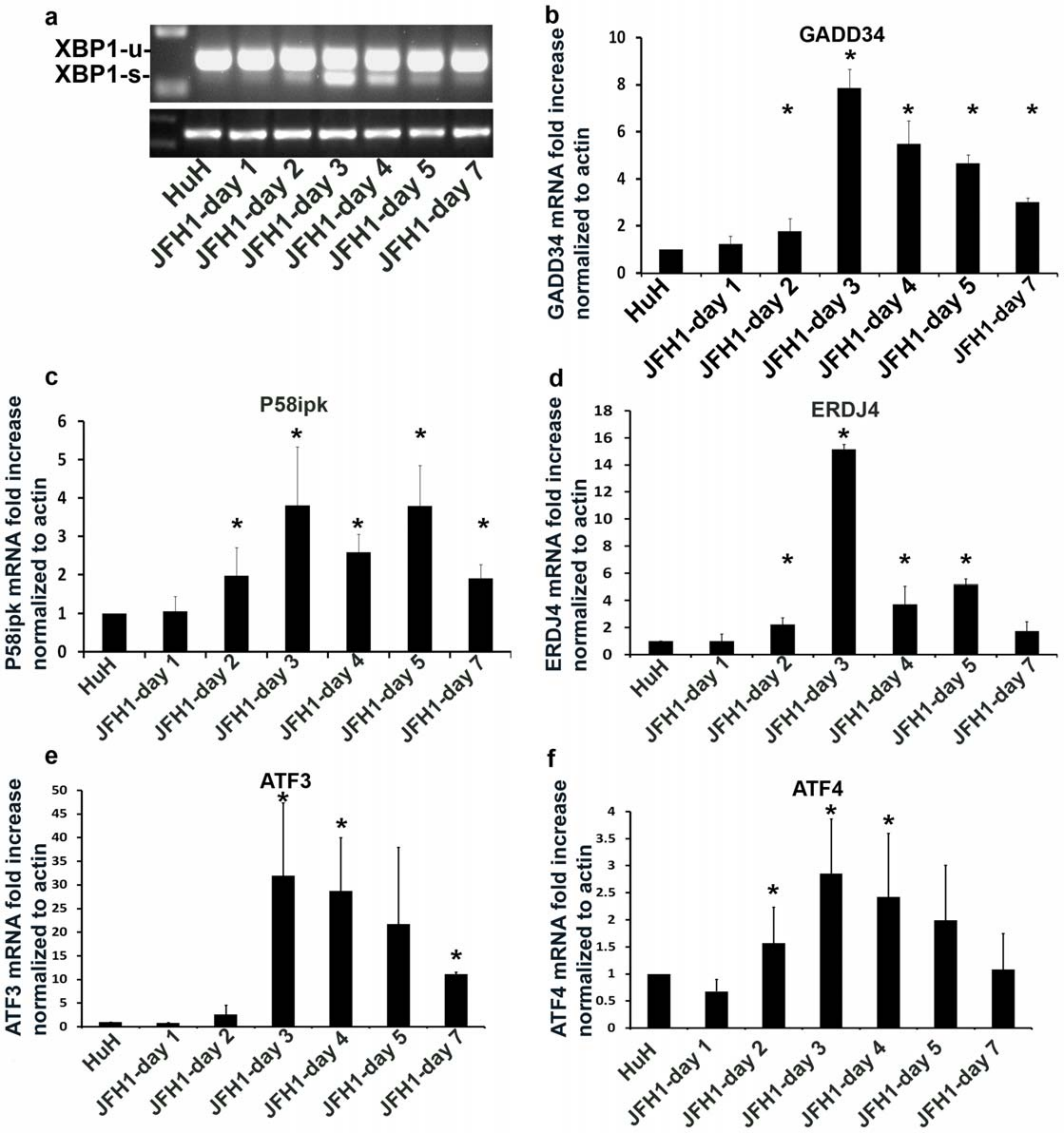
Downstream target genes of the UPR are up-regulated following HCV infection. HuH7.5.1 hepatoma cells were infected with HCV-JFH1. Following infection, cells were harvested and RNA was extracted at the indicated time points. (a) splicing of XBP-1 was measured by RT-PCR. Gene expression levels of (b) GADD34 (c) p58ipk (d) ERDJ4 (e) ATF3 and (f) ATF4 were determined by real-time PCR relative to b-actin. Results are means±S.D. of 3 experiments. * = p<0.05 (ANOVA, one way) versus non-infected cells.

Next, we monitored the time course of UPR induction. The three arms of the UPR were activated simultaneously in a wave-like pattern. Levels of phosphorylated IRE1, cleaved ATF6 and phosphorylated eIF2α, peaked on days 3–5 by 4.13±0.6 fold, 8.3±1.8 fold and 13.6±1.14 fold compared to non-infected cells (Figure 1a, b and c, respectively), in a manner that coincided with the peak viral replication. Phosphorylated eIF2α leads to induction of CHOP and its translocation into the nucleus. Indeed, we observed accumulation of CHOP in the nucleus of HCV infected cells as compared to non-infected controls (Figure 1d). XBP-1 splicing followed a similar wave pattern, which reached a peak at day 3 post infection (Figure 2a). To determine if the IRE1-XBP-1 and PERK-eIF2α arms are transcriptionally active we measured expression of their direct targets by real time PCR. mRNA levels of p58^ipk^ and ERDJ4, ATF3 and ATF4 were up-regulated in a temporal pattern similar to XBP-1 splicing (3.8±1.54, 15.14±0.37, 31.96±15.44 and 2.86±1.01 fold increase compared to non-infected cells, respectively). ATF4 was shown to up-regulate transcription of several UPR targets including that of the growth arrest and DNA damage (GADD)34 gene. GADD34 was shown to promote dephosphorylation of eIF2α via protein phosphatase 1 (PP1c) thereby leading to recovery from translational inhibition in the UPR. We observed a marked increase in GADD34 transcription peaking on day 3 post-infection (8.1±1.2 fold increase compared to non-infected cells) (Figure 2b–f).

ER stress/UPR pathways are interrelated to inflammatory pathways via IRE1/TRAF2. We therefore looked at associated inflammatory pathways with regard to ER stress. We observed a marked increase in the phosphorylation of JNK, peaking on days 3–4 in parallel to the peak of viral replication and the activation of the three arms of the UPR (Figure S3b). These results suggest that inflammatory pathways are activated in tandem to UPR pathways.

We conclude that the IRE1-XBP-1-ERdj4-p58^ipk^and PERK-eIF2α-ATF4-ATF3-CHOP-GADD34 pathways are activated following HCV infection. ATF6 was cleaved, a marker of its activation. Downstream target genes of ATF6 were not investigated in our study.

### HCV infection induces prolonged activation of the UPR

**Figure 3:**
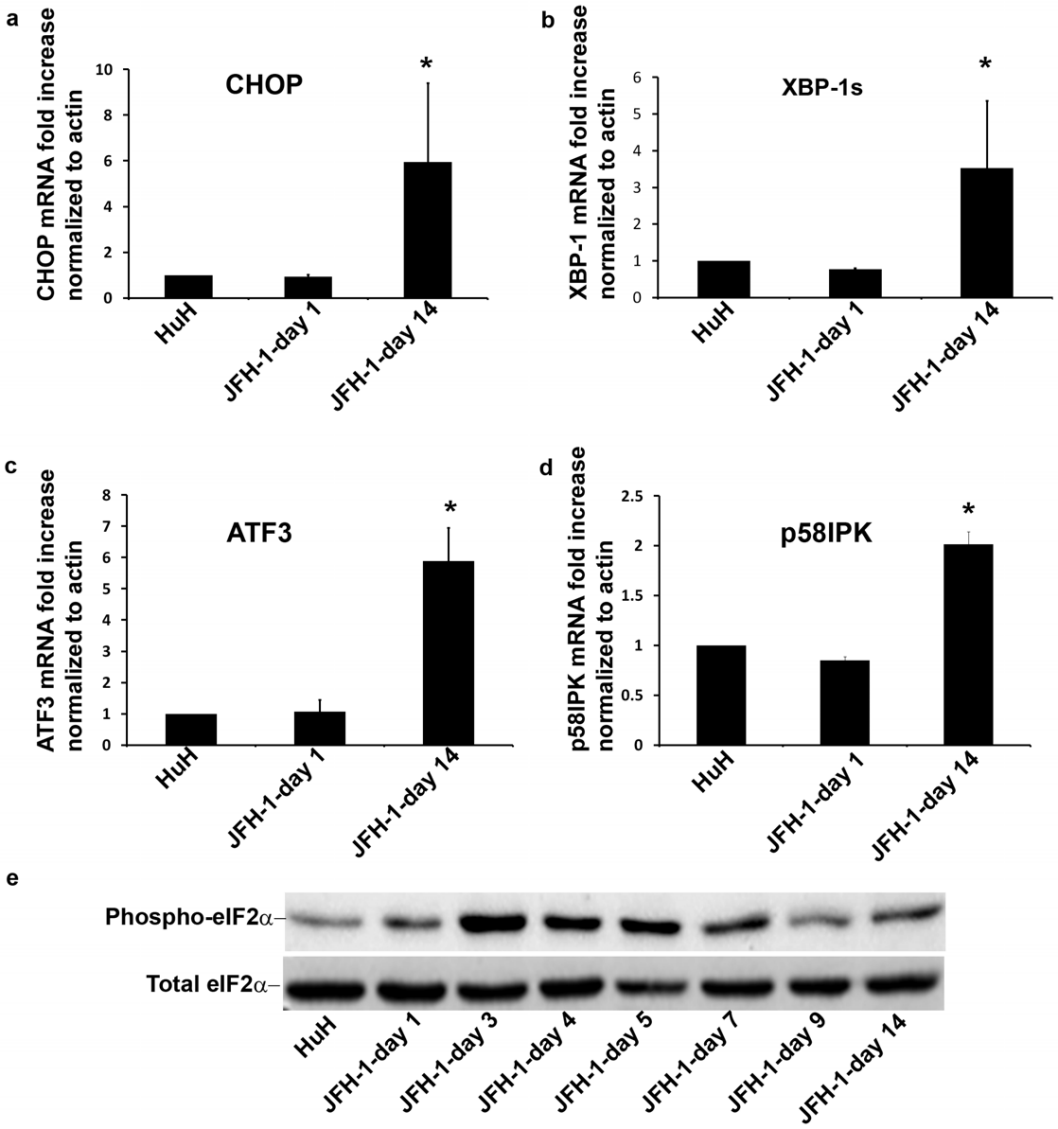
HCV infection induces prolonged activation of the UPR. HCV infected HuH7.5.1 cells were grown in culture for 14 days and compared to non-infected cells and day 1 post-infection cells. Cells were harvested, RNA extracted and gene expression levels were determined by real-time PCR and normalized to b-actin expression. Results are means±S.D. of 3 independent experiments. * = p<0.05 (ANOVA, one way) for day 14 versus non-infected and day 1 infected cells.

Because HCV establishes a chronic infection and since the UPR is activated in the course of infection, we wanted to assess whether HCV induces chronic conditions of ER stress. We analyzed cells 14 days after infection, a time point at which the parameters of HCV infection, such as expression of viral RNA and viral proteins, exhibit steady state levels. We compared by real-time PCR the levels of UPR targets at day 14 to their levels at day 1. We observed that markers of UPR activation did not return to their baseline levels 14 days after infection. Both targets of PERK (CHOP and ATF3) and the IRE1 pathways (spliced XBP-1 and p58^IPK^) were significantly elevated at day 14 relative to day 1 or non infected cells (Figure 3a–d). Similarly, eIF2α phosphorylation remained elevated throughout the infection above baseline (Figure 3e). These results show that HCV infection perturbs the homeostasis of the ER causing a chronic stress, which leads to a sustained activation of the UPR.

### HCV-induced chronic ER stress confers resistance to drug induced UPR activation

**Figure 4:**
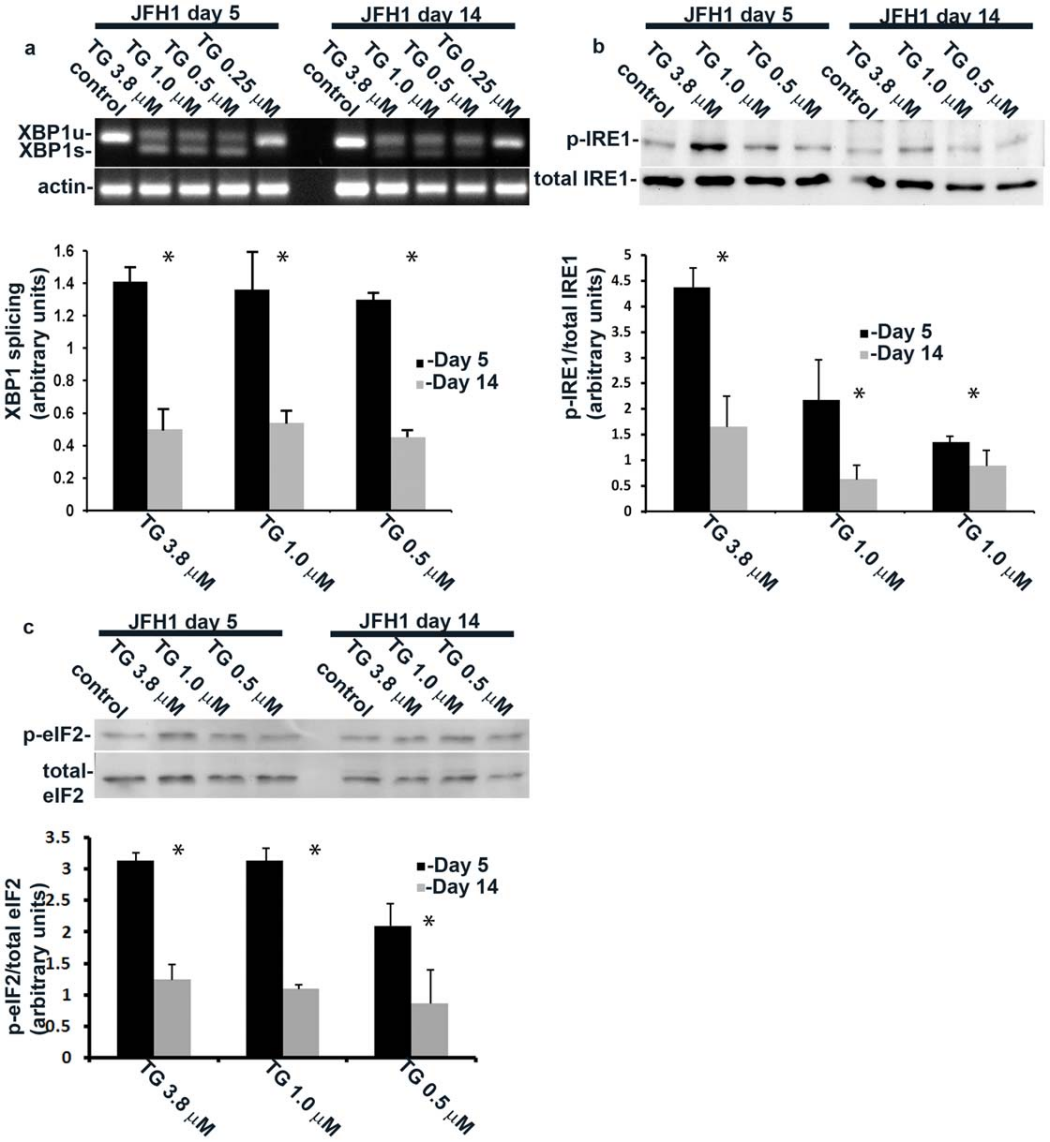
HCV-induced chronic ER stress confers resistance to drug induced UPR. HCV infected cells were stimulated by decreasing doses of thapsigargin at day 5 and at day 14 post infection and the effect of stimulation on (a) XBP-1 splicing or (b) IRE1 and (c) eIF2α phosphorylation was assessed by RT-PCR and immunoblotting respectively. Quantification of the results was obtained from densitometric scanning of the blots (means±S.D.) of 3 experiments. * = p<0.05 (ANOVA, one way) for day 5 versus day 14 post infection.

Artificial induction of chronic ER stress with a sublethal concentration of tunicamycin or thapsigargin causes adaptation to further induction of ER stress [15]. To test whether HCV infection induces a similar adaptation, we treated infected HuH7.5.1 cells at days 5 and 14 with thapsigargin at increasing concentrations and measured the induction of IRE1 and eIF2α phosphorylation, and XBP-1 splicing. Thapsigargin treatment at day 14 caused a markedly attenuated activation of the UPR as demonstrated by reduced IRE1 and eIF2α phosphorylation, as well as diminished XBP-1 splicing compared to day 5 (Figure 4a–c). Our data indicate that HCV-induced chronic ER stress leads to adaptation and reduced activation of the UPR in response to chemical perturbation of protein folding.

### HCV-Tg mice display chronic ER stress, activate UPR genes less efficiently than controls and succumb to ER stress at higher frequency

**Figure 5:**
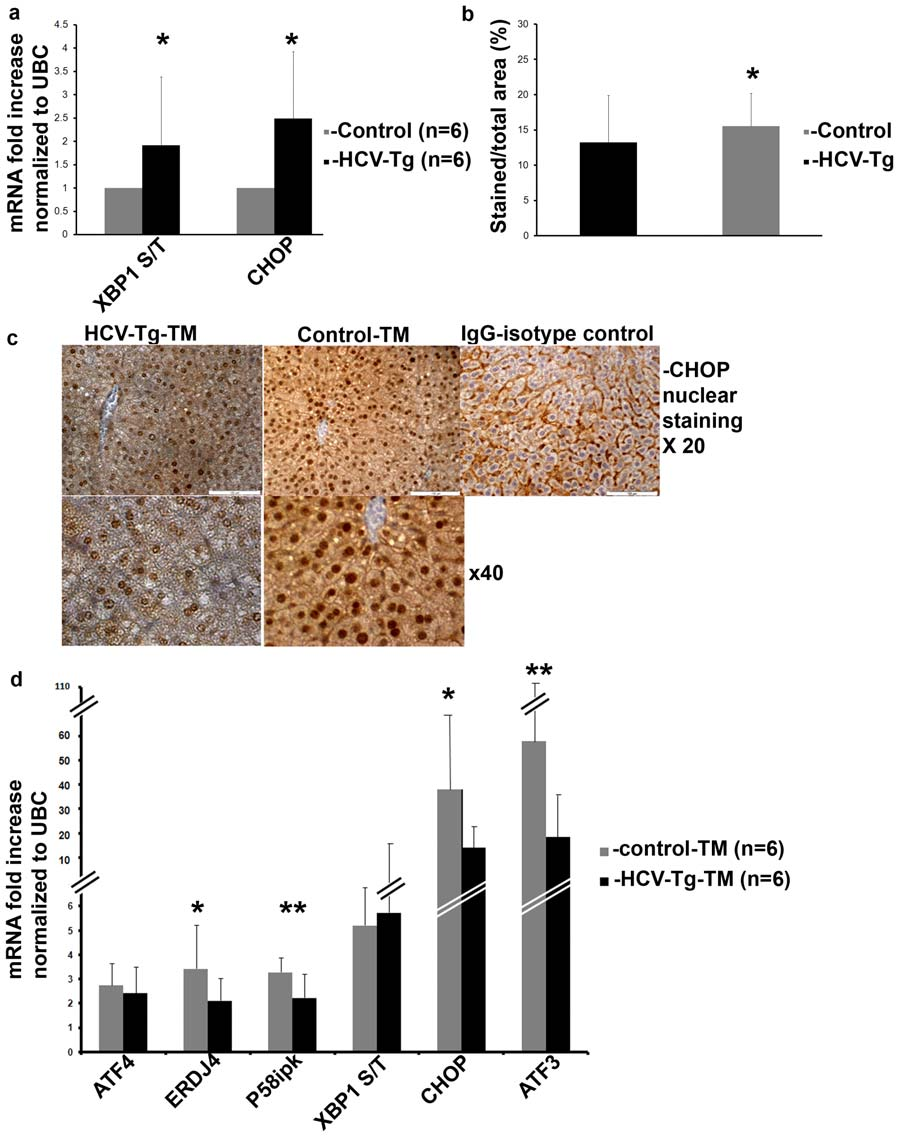
HCV-Tg mice display chronic ER stress and activate UPR genes less efficiently than controls following ER stress induction. Untreated HCV-Tg and control mice were sacrificed, liver excised and RNA was extracted. (a) Expression of the ratio of XBP-1s to XBP-1u (S/T) and CHOP were assessed by real-time PCR. (Panels b, c and d) Mice were treated i.p. with tunicamycin injection of 1 mg/kg every other day (total of 2–3 injections), and were sacrificed 3 days following the last injection. (c) Immunostaining of formalin-fixed livers for CHOP (b) Quantification of stained nuclei was done automatically by visualizing the slides on an Olympus BX61 microscope, photography was performed on Photometric CoolSnap ES and analysis was done using Ariol SL-50 Applied Imaging 3.1.270 software. Untreated mice received 5% dextrose injection.(d) gene expression levels were determined by real-time PCR relative to UBC expression. Results are means±S.D. of 3 independent experiments. *p<0.05, **p<0.01 (ANOVA, one way) for (a) untreated control versus HCV-Tg mice and for (b, d) HCV-Tg control versus HCV-Tg tunicamycin treated mice.

Despite the expression of viral RNA and proteins (Figure S2), HCV-Tg mice exhibit very limited hepatic inflammation. To test whether chronic ER stress develops in the HCV-Tg mice, we compared by real-time PCR the liver expression of UPR genes of HCV-Tg to control animals. At baseline, UPR genes were mildly elevated in HCV-Tg mice livers (ratio of XBP-1 spliced/total and CHOP 1.91±1.4 and 2.48±1.4 respectively compared to controls, p<0.05) (N = 6 mice in each group), suggesting mild but persistent conditions of chronic ER stress (Figure 5a).

To test the development of UPR adaptation in vivo, mice were injected with tunicamycin twice at a day interval. CHOP translocation to the nucleus was decreased in HCV-Tg mice compared to controls (from 15.5±4.6 to 13.2±6.6, % of stained/total area p<0.02) as quantified by an automated cellular scanning quantization system (Figure 5b), and as observed by immunohistochemistry (Figure 5c). consistent with these data, UPR genes elevation was attenuated following tunicamycin injections in HCV-Tg mice compared to wild type controls (3.4±1.1, 3.1±1.0, 35.3±17.1, 56.8±21.3 for ERdj4, p58^ipk^, CHOP and ATF3 respectively p<0.05 for all) (Figure 5d) (N = 6 mice in each group). The attenuation of UPR activation suggests that adaptation to chronic ER stress occurs in-vivo. In accord with these data, when a regimen of three injections was used, we observed higher mortality of HCV-Tg as compared to control-tunicamycin treated mice (HCV-Tg 9/22 (40.9%) vs. wt 3/12(25%)).

Our results strongly suggest that HCV-induced chronic ER stress adaptation compromises the ability to activate the homeostatic cyto-protective response of the UPR in response to a strong ER stress induction.

### HCV suppression by interferon α treatment reverses the host cell adaptation to chronic ER stress

**Figure 6:**
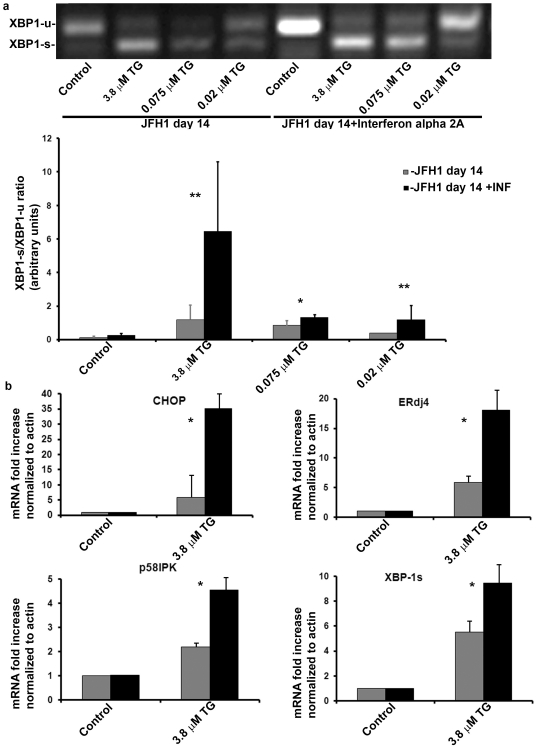
Interferon α treatment reverses the adaptation to chronic ER stress. HCV infected cells were treated with Interferon á 2a (1800 IU/ml were added to cell growth medium and changed daily), or left untreated. On day 14 post infection, cells were stimulated with thapsigargin as indicated. (a) XBP-1 splicing was assessed by RT-PCR. Quantification of the results was obtained from densitometric scanning of the blots (means±S.D.) of 3 experiments. * = p<0.05, **p<0.01 for interferon treated versus non-treated cells. (b) Gene expression levels were determined by real-time PCR and normalized to b-actin expression. Results are means±S.D. of 3 independent experiments. *p<0.05, (ANOVA, one way) for interferon treated versus non-treated cells.

To determine whether ER stress adaptation requires the continuous presence of the virus and to rule out the possibility that viral infection selected for ER stress resistant cells, we suppressed viral replication by interferon α 2a. When ER stress was induced by thapsigargin (Figure 6a), interferon treated cells responded stronger as assessed by enhanced XBP-1 splicing (Figure 6a). Correspondingly, real-time PCR showed a marked increase in gene expression of CHOP, p58^ipk^ and ERdj4 following thapsigargin administration in the interferon treated cells (Figure 6b). Our data show that viral suppression reverses the adaptation and restores UPR responsiveness to ER stress. This indicates an active virus-dependent mechanism of adaptation rather than a passive selection process in the course of culturing the infected cells.

## Discussion

The ability of HCV to evade eradication is mediated in part by manipulation of innate immunity [32], disruption of NF-kB signaling, aberration of cytokine and chemokine secretion [33] and attenuation of interferon response [34]. The role of ER stress in mediating HCV-induced liver damage was recently put under intense scrutiny.

Any increase in the influx of proteins into the ER putatively may elicit conditions of ER stress. Thus, viruses which encode glycoproteins usually provoke ER stress conditions and induce the UPR in the course of infection. While the UPR may play a “mere” cyto-protective role to restore homeostasis following viral infection, evidence accumulated in the context of infection by different viruses strongly suggest that the UPR is utilized by viruses for their own benefit and that it plays a direct role in their cell cycle. For example, under strong ER stress conditions, human cytomegalovirus (HCMV) fails to replicate [35]. It is therefore not surprising that HCMV evolved strategies to minimize ER stress in the course of its replication, for instance by promoting the synthesis of Bip, the master regulator of ER stress [36]. Moreover, other viruses of the herpes family, such as EBV, have usurped the UPR to transcriptionally activate viral genes thereby controlling their lytic cycle [37].

What is the role of UPR in HCV infection? In this respect acute and chronic ER stress may play different and even opposite roles. For example, deletion of XBP-1 in the liver, which confers ER stress, impairs triglyceride synthesis and VLDL secretion [38]. Similar findings were found when GADD34, which neutralizes the PERK pathway of the UPR, was over-expressed in the liver [39]. In contrast, infliction of acute ER stress conditions by tunicamycin injection in various UPR deficient animals induced robust steatosis [40]. Interestingly, severe steatosis is also seen in HCV carriers particularly in genotypes II and III. Taken together, the exact impact of UPR on liver lipid metabolism may be related to the level of UPR activation and its duration.

Our data clearly show that in the course of productive HCV infection all 3 arms of the UPR pathways operate in tandem in a wave-like pattern, which coincides with the replication of JFH1 (Figure 1 and 2). This suggests that ER stress is proportional to the general load of viral proteins. Once replication declines, the cells regain homeostasis, and ER stress decreases but is not terminated (Figures 3 and 5). This feature has not been previously shown.

Our results suggest that HCV, even at low levels of replication, constitutively activates the UPR. This may be due to cooperative activities of various HCV-encoded proteins with respect to their effect on ER physiology. Indeed, the UPR was measured in response to over-expression of multiple HCV proteins such as the structural proteins E1 and E2, core as well as non-structural proteins [16]–[20], [32]. The fact that we observed hyper eIF2α phosphorylation at the chronic phase may actively serve the virus, as E1 and E2 HCV envelope proteins induce eIF2α phosphorylation and repress global translation [17], while maintaining their synthesis in an eIF2α independent fashion [41]. Other viral proteins, such as E2 and NS5A, negate eIF2α phosphorylation [42], [43]. Thus the PERK pathway of the UPR signaling is carefully regulated by the virus to allow optimal protein synthesis for its benefit.

With respect to human pathology, chronic UPR activation may reflect the in vivo situation in HCV carriers. Direct evidence in humans for UPR induction are conflicting as a recent report demonstrates UPR activation in HCV patients long after the initial event of infection [44], while another study showed activation of the three UPR sensors but no induction of UPR-responsive genes [45]. This may be due to the technical difficulty to analyze a small proportion of infected cells amidst vast excess of non-infected hepatocytes.

One of the hallmarks of chronic ER stress is the reduced sensitivity to further induction of the UPR as described for chemically-induced chronic ER stress [15]. We observed a similar phenomenon in HCV infected cells (Figure 4). Hence, the adaptation to chronic stress is not dependent on the mode of stress infliction and should be taken into account in physiological and pathological contexts. Moreover, mice that constitutively express the viral RNA and proteins directed by a liver specific promoter also display characteristics of adaptation (Figure 5). Therefore, the chronic adaptation does not require acute conditions associated with the initial phase of HCV infection. Interestingly, the HCV-Tg mice succumbed to acute tunicamycin-induced ER stress at higher frequency than controls. While this may not be directly related to the handicapped UPR of the transgenic mice, our data is consistent with the fact that expression of viral proteins, even without apparent viral replication, manipulates the UPR in a manner that can be exploited for therapy. To date there are several chemical chaperones, that were shown to regulate ER stress and may potentially act as anti-viral agents. Of interest, HMG-CoA reductase inhibitors (statins) which were recently shown to have anti-HCV properties [46], were also shown to regulate the UPR [47].

Is the adaptation beneficial to the virus? Several studies demonstrate that chronic ER stress alters biological pathways relevant to the viral life cycle, such as apoptosis, intracellular lipid distribution and autophagy. It was shown in HCV expressing cells from HCV-Tg mice, and liver tissue from HCV infected patients, that induction of ER stress resulted in up-regulation of protein phosphatase 2A (PP2A). This led to inhibition of Interferon signaling and decreased degradation of the anti-apoptotic protein, Bcl-2 resulting in increased survival of HCV infected cells [48], [49]. Thus, we propose that chronic ER stress may serve as a novel strategy for manipulation of host defenses by the virus for its benefit. As an additional indication to this hypothesis, viral suppression by interferon treatment reversed the adaptation (Figure 6). In conclusion, we show that HCV induces acute and chronic ER stress and UPR activation resulting in adaptation and reduced response to further stress. Viral eradication resulted in regained sensitivity to ER stress and UPR activation. Our study demonstrates for the first time a biological role of chronic ER stress in a pathogenic context and highlights the ER stress/UPR machinery as a possible anti-viral drug target.

## Supporting Information

Figure S1 JFH1 HCV fully infective cell system: (a) Titration of viral infection was done and TCID50 was calculated as described in the methods. HuH7.5.1 hepatoma cells were infected with the HCV-JFH1 virus. RNA and protein were extracted on the indicated times and assessed for (b) HCV RNA (c) NS5A by real-time PCR and western blotting respectively.
(TIF)

Figure S2 HCV-Tg mice: HCV-Tg and control mice were sacrificed, liver excised and protein extracted and assessed for HCV-Core protein by western blotting.
(TIF)

Figure S3 ER stress and JNK phosphorylation are induced in HCV infected cells: Infected and non-infected HuH7.5.1 cells were grown in parallel under the same conditions of nutrient supply and cell density. Following infection, protein was extracted at the indicated time points. The expression of (a) phospho-IRE1, total IRE1, phospho- eIF2α and total eIF2α and (b) phospho and total JNK were analyzed by western blotting. Actin was used as a loading control.
(TIF)
