# Supplementary figures and images for: HCV Causes Chronic Endoplasmic Reticulum Stress Leading to Adaptation and Interference with the Unfolded Protein Response
Source: PLoS One. 2011 Sep 19;6(9):e24660. doi: 10.1371/journal.pone.0024660 (PMC3176279; doi:10.1371/journal.pone.0024660)

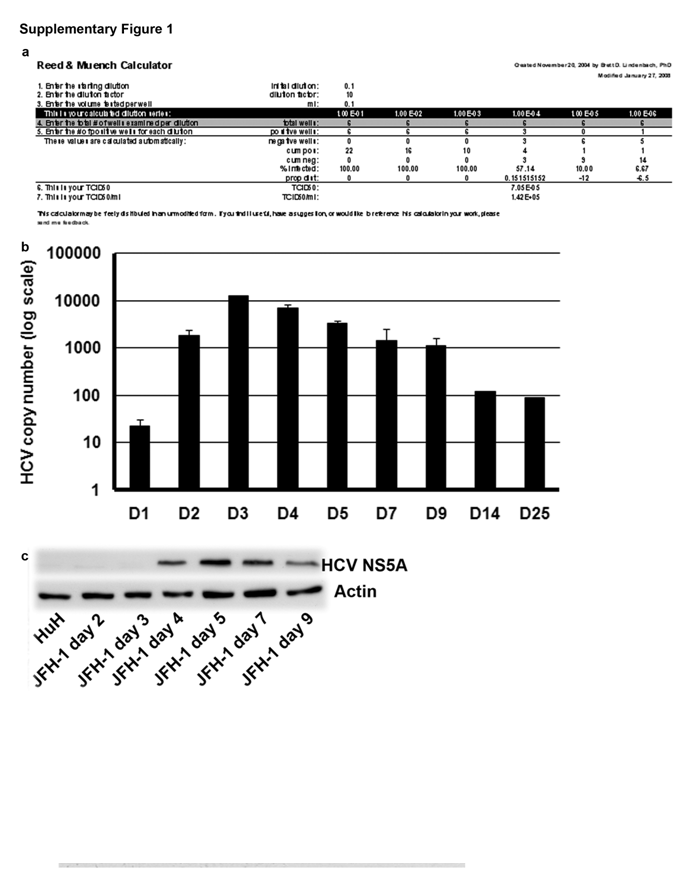

Supplement: Figure S1 — JFH1 HCV fully infective cell system: (a) Titration of viral infection was done and TCID50 was calculated as described in the methods. HuH7.5.1 hepatoma cells were infected with the HCV-JFH1 virus. RNA and protein were extracted on the indicated times and assessed for (b) HCV RNA (c) NS5A by real-time PCR and western blotting respectively. (TIF) [file pone.0024660.s001.tif]

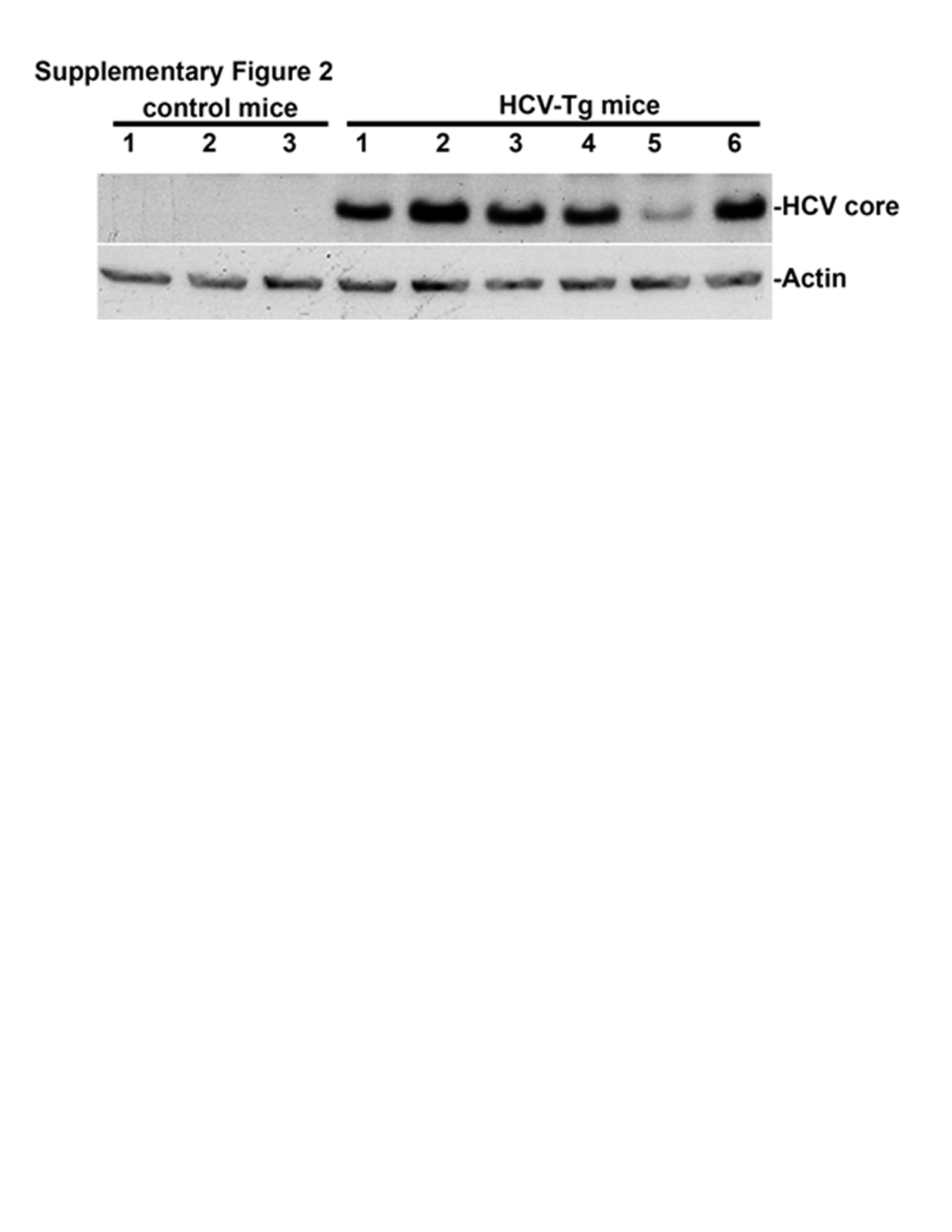

Supplement: Figure S2 — HCV-Tg mice: HCV-Tg and control mice were sacrificed, liver excised and protein extracted and assessed for HCV-Core protein by western blotting. (TIF) [file pone.0024660.s002.tif]

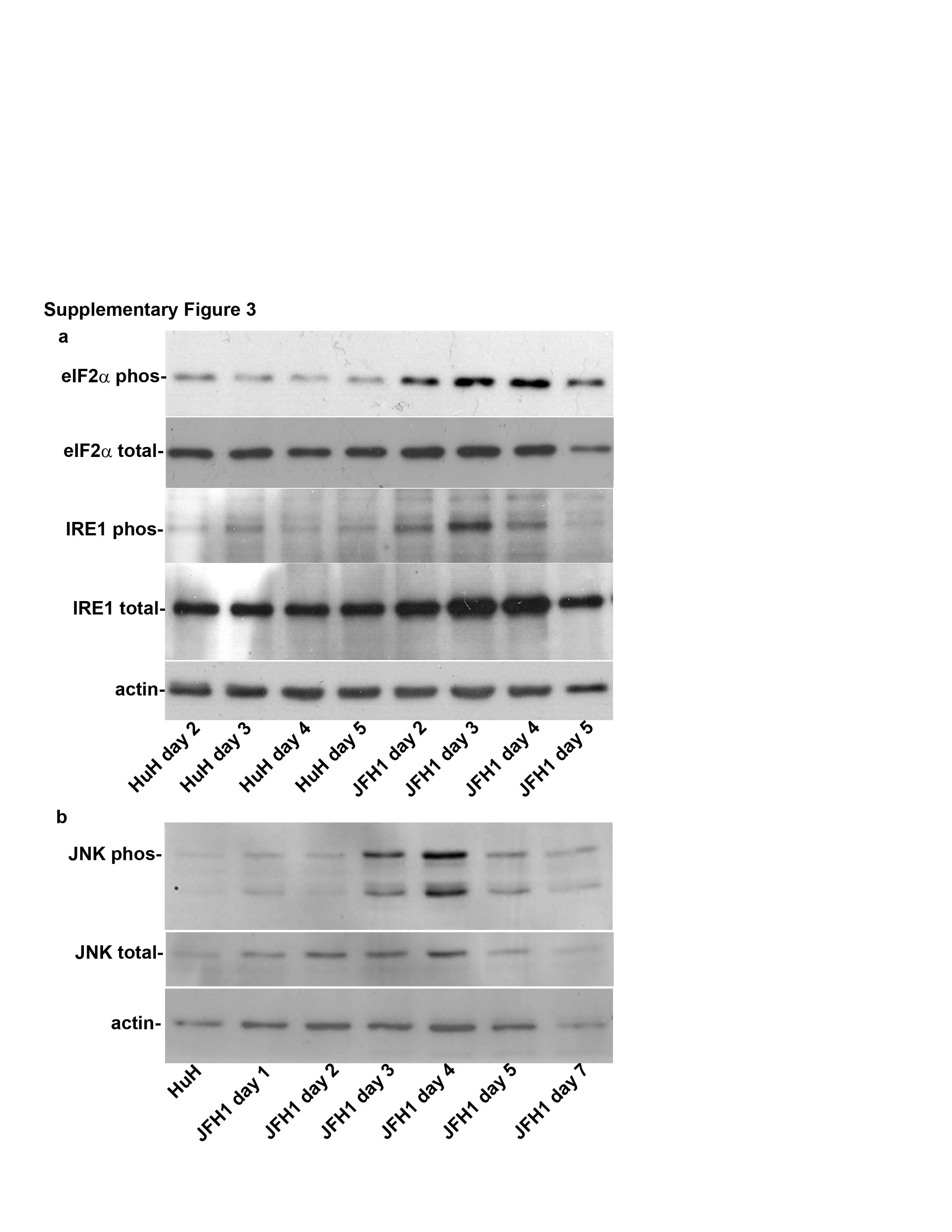

Supplement: Figure S3 — ER stress and JNK phosphorylation are induced in HCV infected cells: Infected and non-infected HuH7.5.1 cells were grown in parallel under the same conditions of nutrient supply and cell density. Following infection, protein was extracted at the indicated time points. The expression of (a) phospho-IRE1, total IRE1, phospho- eIF2α and total eIF2α and (b) phospho and total JNK were analyzed by western blotting. Actin was used as a loading control. (TIF) [file pone.0024660.s003.tif]
